# National Multicenter Study on the Prevalence of Carbapenemase-Producing Enterobacteriaceae in the Post-COVID-19 Era in Argentina: The RECAPT-AR Study

**DOI:** 10.3390/antibiotics13121139

**Published:** 2024-11-27

**Authors:** Mariano Echegorry, Paulina Marchetti, Cristian Sanchez, Laura Olivieri, Diego Faccone, Florencia Martino, Tomas Sarkis Badola, Paola Ceriana, Melina Rapoport, Celeste Lucero, Ezequiel Albornoz, RECAPT-AR Group, Alejandra Corso, Fernando Pasteran

**Affiliations:** Servicio Antimicrobianos, National Reference Laboratory in Antimicrobial Resistant, National Institute of Infectious Diseases (INEI), Administración Nacional de Laboratorios e Institutos de Salud (ANLIS) “Dr. Carlos G Malbrán”, Ave. Velez Sarsfield 563, Buenos Aires City 1281, Argentina; marianoechegorry@gmail.com (M.E.);

**Keywords:** carbapenemase, Enterobacterales, COVID-19, metallo-β-lactamase

## Abstract

The COVID-19 pandemic has exacerbated the global antimicrobial resistance (AMR) crisis. Consequently, it is more urgent than ever to prioritize AMR containment and support countries in improving the detection, characterization, and rapid response to emerging AMR threats. We conducted a prospective, multicenter study to assess the prevalence of carbapenemase-producing Enterobacterales in infectious processes in Argentina during the post-COVID-19 pandemic period and explore therapeutic alternatives for their treatment (RECAPT-AR study). Methods: A total of 182 hospitals participated by submitting Enterobacterales clinical isolates to the National Reference Laboratory (NRL) during the first three weeks of November 2021. Inclusion criteria were defined as an ertapenem MIC ≥ 0.5 mg/L, a zone diameter ≤ 22 mm. Carbapenemase genes and those coding for major extended-spectrum β-lactamases were molecularly characterized using multiplex PCR at the NRL. Antibiotic susceptibility testing followed international standards (CLSI and EUCAST). Results: The NRL analyzed 821 Enterobacterales isolates. Metallo-β-lactamase (MBL, 42.0%) and KPC (39.8%) accounted for 81.8% of carbapenemases, followed by OXA-163 (7.4%), a variant of OXA-48 with additional activity against extended-spectrum cephalosporins, and enzyme combinations (8.3%). These combinations included NDM plus KPC (3.4%), OXA-163 plus KPC (2.4%), and OXA-163 plus NDM (2.1%). *Klebsiella pneumoniae* was the main species recovered, representing 76% of the isolates. According to the carbapenemase classes or combinations, tigecycline exhibited a susceptibility range of 33–83%, fosfomycin 59–81%, colistin 27–78%, and amikacin 17–81%. Ceftazidime-avibactam (CZA) and imipenem-relebactam (IMR) showed 92% and 98% susceptibility against serine carbapenemases, respectively. Meanwhile, aztreonam-avibactam (AZA) exhibited 96–98% susceptibility against all carbapenemase classes. Conclusions: A new epidemiological landscape has emerged, characterized by the equivalent circulation of NDM and KPC. *K. pneumoniae* remains the primary species responsible for their dissemination. The co-production of carbapenemase combinations, particularly KPC plus NDM, was confirmed, mainly in *K. pneumoniae*. High activity was observed for AZA against MBLs and for CZA and IMR against KPC and OXA-163 carbapenemases.

## 1. Introduction

Antimicrobial resistance (AMR), particularly among Gram-negative species, continues to escalate and has been identified as a critical global health threat by the World Health Organization (WHO) [1]. The extensive use of carbapenems to manage multidrug-resistant infections has consequently driven a rise in carbapenem resistance, particularly among challenging pathogens like Enterobacterales, notably *Klebsiella pneumoniae*. Surveillance of AMR is essential at local, national, and global levels to (1) establish effective guidelines for empiric antimicrobial therapy, (2) raise awareness, and (3) limit the spread of AMR.

Data collected between 1997 and 2016 already documented a global upward trend in β-lactam-resistant Enterobacterale species. Latin America has experienced a particularly significant increase in ESBL (Extended-Spectrum β-Lactamase) phenotype rates, with a rise of 22.4%, alongside a notable surge in carbapenem-resistant Enterobacterales, increasing from 0.8% to 6.4%. This escalation has predominantly affected *K. pneumoniae*, which reached a 5% resistance rate by 2008 [2]. In Argentina, similar trends were observed with early reports of carbapenemase-producing Enterobacterales, specifically KPC in 2006 [3] and NDM in 2014 [4]. During that decade, the epidemiology was dominated by KPC producers [5], with *K. pneumoniae* as the main species carrying carbapenemases. Data from Argentina’s National Antimicrobial Resistance Surveillance system (World Health Organization Network, WHONET) shows an annual 2% increase in *K. pneumoniae* carbapenemase prevalence from 2016 to 2019 (https://www.scribd.com/document/754410374/Vigilancia-Nacional-de-La-Resistencia-a-Los-Antimicrobianos-Tendencia-2010-2021-Parcial-Red-WHONET, accessed on 28 October 2024).

Recent studies on carbapenemase epidemiology in Latin America and the Caribbean have documented widespread dissemination of KPC-type carbapenemases in Enterobacterales, now endemic in some countries. Other carbapenemases, like NDM and, to a lesser extent, IMP and VIM, have also been detected [6,7,8].

The SARS-CoV-2 pandemic has substantially exacerbated the AMR crisis, placing immense pressure on healthcare and social care systems. A 2022 CDC report revealed that deaths from antimicrobial-resistant infections exceeded 29,400 in 2020, with nearly 40% of infections acquired in hospitals. The pandemic stretched healthcare resources, resulting in the increased use of medical devices, prolonged patient stays, staffing shortages, and reduced infection control, all contributing to a rise in healthcare-associated infections [9]. Although broad-spectrum antibiotics are ineffective against viruses like SARS-CoV-2, their use has increased dramatically to prevent secondary bacterial infections in severely ill patients, particularly in intensive care settings [10]. As a consequence, a concerning rise in microorganisms with extensively drug-resistant, particularly carbapenem resistance, has emerged. This accelerated spread of multidrug-resistant bacteria and fungi has become particularly evident in intensive care units, where an increase in device-associated infections, especially those linked to central venous catheters and mechanical ventilation, has been observed. In Argentina, as in other regions, this situation is marked by a rise in healthcare-associated infections due to multidrug-resistant bacteria. *K. pneumoniae* carbapenemase-related resistance surged from 20% in 2019 to 30% in 2020, according to Argentina’s WHONET Network during the pandemic (https://www.scribd.com/document/754410374/Vigilancia-Nacional-de-La-Resistencia-a-Los-Antimicrobianos-Tendencia-2010-2021-Parcial-Red-WHONET, accessed on 28 October 2024). In accordance, a study from the Antimicrobial Testing Leadership and Surveillance Programme (ATLAS) examining global geographic patterns of carbapenem-resistant *K. pneumoniae* isolates found that Argentina ranked among the top three countries with the highest rates of carbapenem-resistant *K. pneumoniae* globally in 2020, following only India and Greece [11]. In a multicenter prospective study conducted in Argentina from 2014 to 2020 among patients with hematological malignancies and those undergoing hematopoietic stem cell transplantation, carbapenem resistance rates in *K. pneumoniae* ranged from 18.4% to 26.4% [12]. Reports confirm the concerning trend of increasing carbapenemase-producing organisms in Latin American hospital-acquired infections, with NDM and dual carbapenemase-producing strains (NDM plus KPC) becoming more prevalent annually, while OXA-48-like producers remain rare among Enterobacterales [8]. Following the pandemic’s onset, isolates with KPC plus NDM combinations appeared in Argentina for the first time, compounding the AMR challenge [13,14]. Additionally, Latin America has seen an emergence of carbapenemase-producing Enterobacterales not previously documented and an increasing number of isolates expressing multiple enzymes [15].

In Argentina, systematic reporting on the impacts, as well as the geographic extent of AMR resulting from COVID-19, has been lacking. To address this complex AMR landscape, a national open multicenter prevalence study, the “Argentine Multicenter Study of Prevalence of Carbapenemase-Producing Enterobacterales—the Challenge of Post-Pandemic COVID-19” (RECAPT-AR), was conducted during the first three weeks of November 2021. This study aimed to map the post-COVID-19 epidemiological landscape, characterize circulating carbapenemase resistance mechanisms, evaluate the effectiveness of therapeutic alternatives, and assess the diagnostic capacity for AMR across participating centers.

## 2. Results

### 2.1. Prevalence of Carbapenemases at the National Level

A total of 821 isolates were included. The most abundant species recovered was *K. pneumoniae* (628/821, 76%), followed by Morganellaceae (57/821, 7%), *Enterobacter cloacae* complex (51/821, 6%) and *E. coli* (38/821, 5%). Species and source of isolation of the isolates are depicted in Table 1.

In 801/821, 97.6% of the isolates, at least one carbapenemase gene was confirmed by PCR. MBL enzymes were the most prevalent carbapenemases, accounting for 345/821, 42.0%, followed by KPC, which represented 327/821, 39.8% of the isolates (*p* 0.37). The rest of the isolates harbored OXA-163 (61/821, 7.4%) and carbapenemase combinations (68/82; 8.3%) as follows: NDM plus KPC (28/821; 3.4%), OXA-163 plus KPC (20/821; 2.4%), OXA-163 plus NDM (17/821; 2.1%), and NDM plus KPC plus OXA-163 (3/821, 0.4%). The reminding 20/821, 2.4% corresponded to non-carbapenemase mediated resistance, mostly found among *Serratia marcescens* (3/19, 15.8% vs. 17/802, 2.1% for other spp., *p* 0.00014). The most detected MBL genes was *bla*_NDM_ in 340/345, 98.5% isolates (*p* < 0.00001), followed by *bla*_VIM_ (4/345 1.2%) and *bla*_IMP_ (1/345 0.3%). Table 2 indicates the distribution of carbapenemase genes among the species recovered. *bla*_GES_ and mcr-1 were not detected among the isolates.

Considering the source of isolation, KPC was most frequently recovered from the abdominal tract (50%) and respiratory tract (49%), while MBLs were prevalent in urine (47%) (*p* 0.022). For bloodstream infections, both KPC and MBL were recovered at similar frequency (75/208, 36.1% versus 92/208, 44.2% respectively) (*p* 0.09). Combinations of carbapenemases were recovered at all infection sites. No significant differences were observed in the distribution of resistance mechanisms when analyzing isolates recovered from patients admitted to critical vs. non-critical areas (*p* 0.14).

Regarding the Nationwide distribution of carbapenemases, it was found that KPC was presented in 17/24 provinces. Meanwhile, MBLs were in 13 out of 24. The distribution of OXA-163 enzymes exhibited significant heterogeneity across 14/24 jurisdictions. Multiple carbapenemase producers were identified in only 8 out of 24 jurisdictions. Considering the prevalence at the jurisdictional level, KPC was found to be the most distributed carbapenemase in 11/24 jurisdictions, while MBL was in 8/24 and OXA-163 was in 3/24. Multiple producers remained as low as 5% in 6 out of 8 provinceswhere it was detected, with exceptions in two provinceswith 16% and 26% prevalence.

### 2.2. Secondary ESBL or plasmidic AmpC (plAmpC) Production Among Carbapenemase Producers

ESBL and/or plAmpC were identified in 64.9% of the carbapenemase producers, as follows: 57.0% CTX-M, 5.4% CMY, 0.7% PER and 1.8% harbored a combination (13 isolates with CTX-M plus CMY and 2 with CTX-M plus PER). KPC co-produced CTX-M or CMY in 41% and 1.2% of the isolates, respectively. *bla*_PER_ was predominantly associated with KPC carbapenemase compared to MBL producers (7/327, 2.1% vs. 1/345, 0.3%, respectively) (*p* 0.0271). Conversely, 86.4% of MBL producers exhibited secondary ESBL and/or plAmpC enzymes, with 73.3%, 9.3%, and 3.8% of the isolates harboring CTX-M, CMY-type or enzyme combinations (12 CTX-M plus CMY and 1 CTXM plus PER), respectively. These additional resistant mechanisms in MBL producers impacted the availability of aztreonam as a therapeutic option (see below). Regarding OXA-163 isolates, secondary CTX-M or CMY was confirmed in 63.9% and 6.4% of the isolates, respectively. Multiple carbapenemase producers co-harbored CTX-M and CMY in 46% and 6% of the isolates, respectively. Considering the bacterial species, the highest rates of secondary ESBL or plAmpC were found in 69.1% of *K. pneumoniae*, 68.4% of *E. coli*, 60.8% of *Enterobacter* spp., and 43.9% of Morganellaceae. Notably, carbapenemase-producing Morganellaceae showed a strong association with CMY (14/57, 24.6%). The primary mechanism associated with carbapenemases was CTX-M, particularly in conjunction with MBL (266/345, 77%) and observed primarily in *K. pneumoniae* (421/628, 67%) (*p* < 0.00001). Table 2 indicates the distributions of secondary ESBL and/or plAmpC genes among the species recovered.

### 2.3. Susceptibility Profile of Carbapenemase Producers

Table 3 shows the susceptibility profile against selected antibiotics for the carbapenemase classes identified in this work. Aztreonam avibactam (AZA) demonstrated the highest activity against the carbapenemase collection (789/819, 96.3% susceptible according to EUCAST and 799/819, 97.6% by CLSI) (*p* < 0.00001), followed by cefiderocol (575/791, 72.7%—658/791, 83.2% susceptible, respectively), fosfomycin (548/767, 71.4% susceptible) and tigecycline (481/732, 65.7% susceptible). Overall susceptibility to CZA was as low as 416/820, 50.7% due to the high prevalence of MBL recovered. The same applied to imipenem relebactam (IMR).

Considering the KPC group, CZA, IMR, AZA, and cefiderocol displayed equivalent activities (97.2%, 98.2–98.5%, 97.8–99.4%, and 93.8–97.2%, respectively) (*p* 0.61), being the most active drugs for this group. No cross-resistance between CZA and IMR was observed for KPC-type enzymes. Older drugs, such as colistin, tigecycline, fosfomycin, and amikacin, displayed susceptibilities between 66.3 and 71.1% (Table 3).

For MBLs, only AZA was uniformly active against these isolates (333/345, 96.5% susceptible by CLSI and 332/345, 96.2% susceptible by EUCAST) (*p* < 0.00001). Cefiderocol (175/342, 51.2% susceptible by EUCAST—236/342, 69.0% by CLSI) (*p* < 0.0001), colistin (194/325, 59.7% susceptible) (*p* 0.0151), amikacin (57/343, 16.6% susceptible) and tigecycline (188/291, 64.6% susceptible) (*p* < 0.0001) were significantly less active against MBL respect to KPC, while fosfomycin (221/309, 71.5% susceptible) (*p* 0.87) resulted equivalent (Table 3).

Patients with severe infections and with a lack of access to new antimicrobials often necessitate treatment with vintage drug combinations. In this regard, the most effective combination for KPC-type enzymes was fosfomycin plus colistin (51.4% susceptible), fosfomycin plus amikacin (49.4% susceptible), and fosfomycin plus tigecycline (48.1% susceptible). These outperformed combinations like tigecycline plus colistin (43.9% susceptible) and tigecycline plus amikacin (42.1% susceptible) (*p* 0.01878). For MBL-type enzymes, the reduced individual rates susceptibilities to amikacin, colistin, and tigecycline impacted when analyzed in combinations: the most effective ones were fosfomycin plus tigecycline (49.1% susceptible), fosfomycin plus colistin (47.4% susceptible) and tigecycline plus colistin (*p* 0.73).

Considering the OXA-163 producers, CZA (95.1%), IMR (95.1% susceptible by EUCAST-91.8% by CLSI) and cefiderocol (89.8–96.6%, respectively) were highly active, as observed for KPC (*p* 0.14). A single isolate presented cross-resistance between CZA and IMR (1/61, 1.6%). AZA demonstrated slightly lower efficacy against OXA-163 (85.2% susceptible by EUCAST-91.8% by CLSI) compared to KPC, likely due to the high hydrolytic activity of this oxacillinase variant against monobactams. This was particularly observed in *E. coli* with an AZA susceptibility of less than 80%, resulting from an active outbreak in a single hospital where this carbapenemase was involved (Table 4). Older drugs, such as colistin, tigecycline, fosfomycin, and amikacin, displayed susceptibilities between 51.7 and 78% (Table 3).

Regarding multiple carbapenemase producers, all isolates showed susceptibility to AZA highly superior to older drugs as colistin, tigecycline, fosfomycin, and amikacin (26.7–83.3% susceptible) (*p* < 0.0001) (Table 3).

Aztreonam, a well-known option for MBL producers, exhibited only 13.3% (EUCAST) or 16.2% (CLSI) susceptibility, with notable species-associated differences such as the Morganellaceae which exhibited 76.9% (EUCAST) or 89.7% (CLSI) susceptibility vs. non-Morganellaceae with 5.2–6.9% susceptibility (*p* < 0.0001).

Morganellaceae demonstrated lower susceptibility to fosfomycin (38.2%) and CZA (15.8%) compared to other species (74.1% and 53.3%, respectively) (*p* < 0.0001). Additionally, susceptibility to colistin varied significantly by species, with *Enterobacter and E. coli* showing higher susceptibility compared to other species. Of note, *E. coli* was the only species that showed almost uniform susceptibility to tigecycline. As mentioned, the non-susceptibility to CZA and IMR paralleled the prevalence of MBL in each bacterial species (Table 4).

### 2.4. Performance of Participating Hospitals for Carbapenemase Detection

Information regarding the carbapenem-resistant mechanism was available for further analysis in 800 out of the 821 isolates. All 800 isolates met criteria indicative of suspicion of carbapenemase production as per the NRL algorithm.

The total agreement between the mechanism inferred by participants and the genotype confirmed at the NRL reached 89.5%. KPC had the highest agreement (96.0%), followed by MBL (93.5%) (*p* 0.16). OXA-163 agreement detection was 90.0% despite the inherent diagnostic challenges associated with this type of enzyme. The distribution of errors was as follows: (i) MISS error: 23/800 (2.9%) strains were reported as belonging to a carbapenemase class different from that confirmed in the NRL, with the most common error being the report of MBL strains as KPC producers (6/23) and the report of KPC strains as MBL producers (5/23); (ii) SUB error: in 27/800 (3.4%) strains, a carbapenemase combination was not reported in dual producers, with the most common error being the no report of OXA among 13/27 isolates with KPC plus OXA and in 6/27 isolates with NDM plus OXA, followed by the miss-identification of one of the carbapenemases in KPC plus NDM dual producers (7/29); (iii) An OVER error was observed in 34 out of 800 strains (4.3%), where an additional carbapenemase was reported beyond what was detected in the genotype. The most frequent error occurred among MBL producers, with 12 out of 34 isolates being reported as dual producers at the local level. This was followed by the erroneous identification of a carbapenemase in carbapenem-resistant, non-carbapenemase-producing isolates.

## 3. Discussion

In this national point of prevalence study, we forecast the landscape of carbapenemase-producing Enterobacterales in Argentina in the immediate post-COVID-19 scenario. This investigation seeks to demonstrate the increase in carbapenem resistance rates, the changes in the epidemiology of carbapenemase producers, and the emergence and dissemination of multiple carbapenemase producers.

Through this study, a change in the paradigm of circulating carbapenemases could be observed: a significant increase in the prevalence of MBL, surpassing KPC for the first time since carbapenemase NDM was first reported [4]. The findings of this study position Argentina among the countries where NDM is now the predominant carbapenemase detected in the post-COVID era, confirming a significant rise in the prevalence of these carbapenemases, which have already been reported across all continents [16,17]. In contrast, this trend is not observed globally; recent surveillance reports on carbapenemase epidemiology in Europe, Latin America, and the Caribbean indicate that KPC-type carbapenemases remain the most prevalent among Enterobacterales in these regions, with their spread reaching endemic levels in certain countries [6,7,8,18,19]

NDM is now the predominant MBL enzyme in our country. This rise in NDM prevalence is linked to *K. pneumoniae* isolates, particularly those from urinary tract infections, and could likely be influenced in part by the increased use of CZA due to the endemic presence of KPC [20]. NDM was the primary carbapenemase in 8 of the country’s 24 provinces, including those with the most demographics.

As reported previously, *K. pneumoniae* continues to be the primary host for carbapenemase dissemination, with a parallel increase in NDM detection [2,15,21]. These findings align with global trends of MBL-type enzyme proliferation and establishment in species already spreading carbapenemases [2,7,15]. In this study, we also observed that NDM is the main carbapenemase in species such as *Citrobacter freundii*, *K. aerogenes, K. oxytoca*, and the Morganellaceae family, albeit with a much lower incidence than in *Klebsiella*. Unlike global trends, KPC remains the main carbapenemase in the *E. cloacae* complex and *S. marcescens* isolates [2,21].

A notable characteristic of the Latin American region, also confirmed in this study for Argentina, is the low prevalence of OXA-48-like enzymes [6,7,8]. In our survey, this carbapenemase had a prevalence of 7.3%, with a non-significant increase compared to previous periods, becoming the third most common carbapenemase of interest in our country. Interestingly, a unique allelic subfamily, OXA-163, represented all isolates within this group. This OXA variant shows enhanced cephalosporin hydrolysis and weaker activity on carbapenems.

Redundant carbapenemase-producing bacteria, which carry double or multiple carbapenemases, represent a new and concerning phenomenon first observed during the COVID-19 pandemic. This study confirmed the persistent circulation of *K. pneumoniae* strains co-producing KPC and NDM carbapenemases following their initial detection during the pandemic [14]. A recent review suggests that the rise of multi-carbapenemase producers may be linked to an accumulation of non-β-lactam resistance mechanisms, as the presence of additional β-lactamases confers limited extra protection against these antibiotics [22]. In our study, multi-carbapenemase producers, especially those co-producing NDM and OXA-163, showed higher levels of co-resistance to non-β-lactam antibiotics. However, the small sample size limits the statistical power of this finding.

We also noted that 65% of the isolates harbored secondary β-lactamases, such as an ESBL or plAmpC, with CTX-M being the most prevalent secondary mechanism across all species, except for Morganellaceae, where CMY predominated. Moreover, CMY showed a strong association with MBL-type enzymes, particularly in *E. coli* isolates and Morganellaceae, surpassing frequencies observed in other species. A significant disparity was observed in the prevalence of these secondary mechanisms, being more prevalent among NDM than KPC producers. This enzymatic profile further narrows therapeutic options, particularly concerning aztreonam susceptibility in Argentina, aligning with previous findings from Latin America that report the lowest susceptibility rates in our country [23]. The exception was the Morganellaceae family, which demonstrated high susceptibility to aztreonam, which contrasts with other species. However, it is crucial to highlight that this susceptibility phenotype does not consistently correlate with the detected genotype. In 40% of *Providencia* spp. and nearly 50% of *Proteus* spp. active resistance mechanisms against monobactams were identified. Notably, the presence of CMY cephalosporinase in the Morganellaceae family elevated aztreonam MICs surpassing the EUCAST susceptible cutoff-point (≤1 mg/L), though still within the CLSI proposed susceptible breakpoint (≤4 mg/L), suggesting that EUCAST standard would be more appropriate for target secondary mechanisms in this family. This highlights the critical need for microbiologists to conduct thorough studies on these species and actively investigate their resistance mechanisms. We emphasize the urgent need to identify plAmpC mechanisms among MBL-producing Morganellaceae to prevent the inappropriate use of monobactams in monotherapy.

Susceptibility to older drugs, typically used in combination therapies for Enterobacterales producing carbapenemases, demonstrated limited effectiveness against these contemporary isolates. In our study, tigecycline exhibited susceptibility rates of 65.7% (33.3% to 83.3% according to carbapenemases class or combinations), fosfomycin 71.4% (58.9–81%), colistin 64.2% (26.7–78%) and amikacin 43.3% (16.6–81%). Recent recommendations advocate for the primary use of novel agents such as CZA or IMR against serine carbapenemases and AZA or cefiderocol for MBL infections [24]. Consistent with these recommendations, our study demonstrated sustained efficacy of these last-line treatment options against serine carbapenemase, with susceptibility rates ranging from 93.3% to 98.7% for IMR and 90% to 97.5% for CZA, in alignment with recent data from global surveillance programs, which also include isolates from Latin America [25,26,27]. Notably, in this study, no co-resistance to CZA and IMR was observed among serine carbapenemase producers.

AZA was the most uniformly active drug against all classes of carbapenemases classes or combinations. These results align with previous studies showing similarly high efficacy of AZA against major carbapenemase classes, with 97.8–99.4% susceptibility reported for KPC, 85.0–91.7% for OXA-48-like, 96.2–96.5% for MBL producers, and 100% for dual producers [23,28,29,30,31]. These findings underscore the importance of this new agent in the management of multidrug-resistant infections, especially MBL producers, highlighting their critical role in improving treatment outcomes and combating antimicrobial resistance globally [32,33].

Cefiderocol is a unique catechol-siderophore cephalosporin approved in Europe for the treatment of infections caused by aerobic Gram-negative organisms in adults with limited treatment options [34]. Its structure and “Trojan Horse” mechanism of bacterial cell entry provide enhanced stability against a wide range of β-lactamases, including MBLs, allowing cefiderocol to maintain its activity broadly [35,36]. It is the second preferred choice for NDM and other MBL-producing Enterobacteriaceae, demonstrating high clinical efficacy [37]. Despite initial reports indicating very high susceptibility rates globally, non-susceptibility to cefiderocol appears more common in carbapenem-resistant Gram-negative pathogens when applying the more stringent breakpoints proposed by EUCAST [38]. Our study yields results comparable to a global meta-analysis, which identified several β-lactamases and other mechanisms leading to higher MICs of cefiderocol, particularly NDM [39]. The mechanisms of resistance to cefiderocol in this report are still under investigation.

Proper detection of carbapenemase-producing Enterobacterales is essential for preventing their spread and ensuring effective treatment. Adapting screening methods to account for emerging resistance mechanisms remains key, as proposed [40]. In this study, microbiology labs demonstrated high accuracy (89.5% concordance) despite the challenges of diverse carbapenemase families and pandemic-related healthcare pressures. The greatest diagnostic challenge involved double producers, especially OXA-48-like co-productions, lacking phenotypic alerts. However, KPC plus NDM detection showed only a minor loss (0.8%), likely due to early phenotypic guidance from the NRL [14]. Potential errors may also stem from mixed populations or genetic material loss during sample transport. Laboratories were able to evaluate their performance by comparing their results with those returned in this study, detect errors in procedures, and implement corrective measures accordingly.

The high mortality rate linked to carbapenemase-producing Enterobacterales infections in Latin America is particularly alarming, with some of the world’s highest rates observed in this region [41]. In Argentina, a prospective multicenter study investigating the clinical impact of bloodstream infections caused by carbapenem-resistant organisms (EMBARCAR) found a 49% fatality rate in the *K. pneumoniae* subgroup, increasing to 52% specifically among NDM producers, the predominant carbapenemase in the study [42]. Our research highlights a shifting epidemiological landscape, with both NDM and KPC carbapenemases now circulating at equivalent and high levels, with *K. pneumoniae* as the leading disseminator. These findings are essential to improving antibiotic susceptibility management and patient outcomes in order to curve the impressive mortality rates in our country. Addressing the unique resistance patterns within various regions is crucial for optimizing treatment strategies and containing the spread of multidrug-resistant pathogens.

A recent study analyzes the global burden of AMR from 1990 to 2021, with projections extending to 2050, underscoring AMR as an escalating public health threat. The study reveals marked regional disparities, with the highest AMR mortality rates for 2050 expected in South Asia and Latin America, painting a bleak outlook for Argentina in particular. For Latin America, substantial improvements could arise from strengthened surveillance systems and infection prevention measures and improved access to effective antibiotics [43]. Our study contributes to advancements in understanding antibiotic susceptibility in the face of evolving threats, with direct benefits for patient care and valuable insights into the updated epidemiological landscape. It also provides essential baseline information to address barriers that low- and middle-income countries face in accessing new antibiotics. Without closing this access and diagnostics gap, consequences could be severe, including higher mortality rates and accelerated dissemination of resistant pathogens [44].

This study has several limitations: (1) the study period may not capture seasonal or temporal variability in the prevalence of these organisms; (2) most participating hospitals were located in the country’s most densely populated areas; (3) although Argentina has approx. 300 high-complexity hospitals, only half of which participated by submitting strains for this survey. While the data show trends consistent with those in other countries, the findings may not be fully generalizable to all healthcare institutions nationwide, and (4) susceptibility to classical antimicrobial agents was assessed locally. Despite high diagnostic efficiency, some uncontrolled trends could remain.

## 4. Materials and Methods

### 4.1. Study Design and Isolates

The RECAPT-AR is a prospective, open multicenter study. A total of 182 hospitals, representing all 24 jurisdictions in the country, participating in the National Surveillance Network for AMR (WHONET-AR Network) and/or the National Quality Assurance Program for Bacteriology, coordinated by the NRL, were enrolled in the study.

The RECAPT-AR was conducted from the 1st to the 21st November 2021. The enrolled institution committed to submit clinical specimens of Enterobacterales isolates to the NRL according to the following inclusion criteria: clinical isolates displaying either an MIC to ertapenem of ≥0.5 mg/L or halo of ≤22 mm or a positive PCR or immune chromatography for specific carbapenemase targets KPC, NDM, OXA-48-like, IMP, or/and VIM. Only a single isolate per patient (first infectious episode) was included. Isolates from surveillance studies were excluded [45]. Isolates were collected based on taxonomic definitions and CDC criteria for infectious diseases [46].

Participating hospital and patient personal information were de-identified and renamed by a preestablished three-letter code. An electronic Google form was requested for each isolate, including a single form for each qualifying strain. The requested information included patient age and sex, bacterial species, site of isolation, and susceptibility profile at local laboratories for all tested antibiotics and the reported carbapenem-resistance mechanism based on available local resources. The collected isolates meeting the inclusion criteria were sent to the National Reference Laboratory (NRL) by December 2021, accompanied by a printed card containing consolidated data.

Hospitals were requested to complete a second online Google form, the Epidemiological Data Form, detailing the distribution of Enterobacterales during the survey period. This form included information on the total number of Enterobacterales recovered and the species distribution. Duplicate samples were excluded.

### 4.2. Reception at the NRL

During the survey period, 824 Enterobacterales isolates meeting the inclusion criteria were received from 150 institutions across the country. The remaining 32 Institutions declared no isolates meeting the inclusion criteria. In this survey, among the one hundred and fifty hospitals that submitted isolates to the NRL, four hospitals provided 20 or more isolates, eight hospitals submitted between 15 and 19 isolates, fifteen hospitals contributed between 10 and 14 isolates, and twenty-eight hospitals sent between 5 and 9 isolates. The remaining ninety-five hospitals each submitted fewer than 5 isolates.

Isolates were subcultured in CHROMagar™ mSuperCARBA™ medium (CHROMagar, Paris, France) to check purity [47]. Species identification was confirmed by mass spectrometry (MALDI-TOF—MALDI Biotyper^®^ 3.0, Bruker, Billerica, MA, USA). Polymicrobial isolates suspected of contamination were excluded (n = 3). Ten isolates initially not growing on CHROMagar™ mSuperCARBA™ were subcultered on trypticsoy agar, and a minimal antibiogram was performed on Mueller Hinton Agar (MHA, Difco, Becton Dickinson, Franklin Lakes, NJ, USA) against piperacillin-tazobactam, ertapenem, meropenem, and ceftazidime-avibactam (CZA) disks (Becton Dickinson) for the recovery of the carbapenemase-producing isolate submitted. A total of 821 isolates were ultimately available for inclusion in the study.

Completeness rates for the electronic Google forms submitted by participants were as follows: 94% for filiatory data, 94% for microbiological data, 92% for susceptibility data, and 88% for epidemiological data.

### 4.3. Molecular Characterization of the Isolates

Molecular testing involved DNA extraction from viable isolates through boiling, followed by multiplex PCR to characterize prevalent carbapenemase genes: *bla*_KPC_, *bla*_NDM_, *bla*_VIM_, *bla*_IMP_, and *bla*_OXA-48-like_. Additional isolates were tested by multiplex PCR-targeted ESBLs (*bla*_CTX-M_, *bla*_PER_) and plAmpC (*bla*_CMY_) and monoplex PCR-targeted for mcr-1 and *bla*_GES_ [14,48,49].

Isolates with a positive result for *bla*_OXA-48-like_ were further characterized utilizing a monoplex PCR assay specific for the OXA-163 allelic variant. PCR products were analyzed through 1% agarose gel (BioRad, Hercules, CA, USA) electrophoresis with Sybr Safe (Thermo Fisher Scientific, Waltham, MA, USA) [14].

### 4.4. Antibiotic Susceptibility Testing (AST)

AST was determined by the agar dilution MIC method (Mueller–Hinton agar from Difco, Becton Dickinson), following CLSI recommendations, against aztreonam (Richet, CABA, Argentina), aztreonam avibactam (Molekula LTD, Darlington, UK), CZA (ceftazidime from Sigma, St. Louis, MO, USA), and imipenem relebactam (Advance ChemBlock Inc., Hayward, CA, USA), using a fixed inhibitor concentration of 4 µg/mL for all β-lactam/β-lactamase inhibitor combinations [50]. Reference strains included *Pseudomonas aeruginosa* ATCC 27853, *Escherichia coli* ATCC 25922, *K. pneumoniae* ATCC BAA1705, and *K. pneumoniae* ATCC 700603, served as controls. As no breakpoint points were available for AZA at the time of the study, we used the breakpoint defined for aztreonam by EUCAST (≤1 mg/L, susceptible) and CLSI (≤4 mg/L, susceptible) [38,50].

Susceptibility to other agents was defined by the data provided in the electronic Data Form submitted by the Institutions. All antibiotics were tested and interpreted using the CLSI breakpoint available at the time of the study, except for colistin and fosfomycin, and tigecycline, where EUCAST and FDA definitions were applied, respectively [38,51]. As no harmonized breakpoints between EUCAST and CLSI were defined for IMR, we categorized the isolates in the present work with both cutoff points [38,50].

A selection of 791 isolates were evaluated for susceptibility to cefiderocol (Shionogi, Osaka, Japan) using broth microdilution (BMD) in iron-depleted cation-adjusted Mueller–Hinton broth (Difco) (prepared according to recommendations by CLSI or EUCAST). Cefiderocol susceptibility was not evaluated in the carbapenem-resistant, non-carbapenemase-producing group. We used both the breakpoint defined for cefiderocol by EUCAST (≤2 mg/L, susceptible) and CLSI (≤4 mg/L, susceptible) for isolate categorization [38,50].

**Figure 1:**
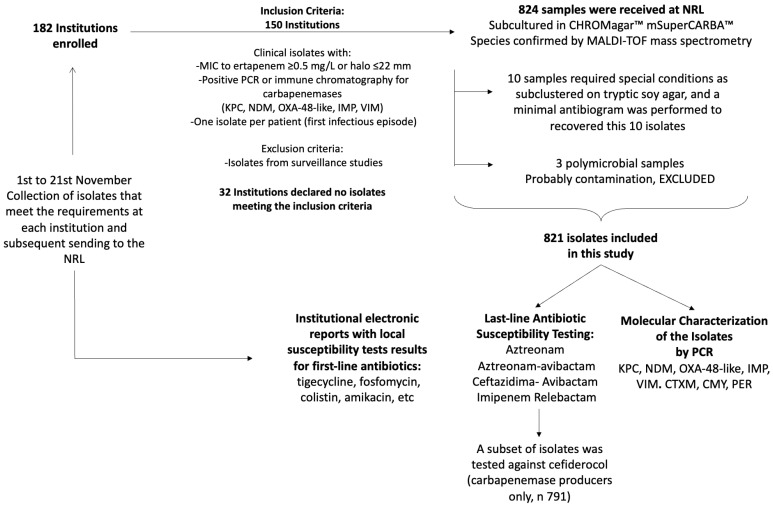
Schematic representation of the workflow, number of isolates, and tests performed.

A schematic representation of the workflow, number of isolates, and tests performed is shown in Figure 1.

### 4.5. Performance of Local Laboratories for Carbapenemase Identification

To screen isolates using NRL algorithms, participating laboratories employed disk diffusion (25.3%) and/or automated systems, including Vitek 2 Compact (Biomerieux, Marcy-l’Étoile, France; 38%) and Phoenix BD (Becton-Dickinson, 36.7%). Suspected isolates were confirmed using at least one of the following methods: 372 μg EDTA and 300 μg 3-amino-phenyl-boronic acid disks (Laboratorios Britania, CABA, Argentina; 149/150, 99.3%), colorimetric assays (96/150, 64%), carbapenem inactivation method (10/150, 6.7%), or molecular or immunochromatographic methods (82/150, 54.6%).

We compared the results of molecular characterization obtained at the NRL with the carbapenemase class reported by local laboratories. Participants reported microbiological information, including phenotypic tests for carbapenemase detection and the inferred carbapenemase classes. We defined the following categories of error: (1) miss-classification (MISS)—discrepant carbapenemase class between participant and the genotype at the NRL; (2) underestimation of one of the carbapenemases harbored by strains with multiple carbapenemase genes (UNDER), and (3) overestimation of carbapenemases (OVER) defined as a local report of more than one carbapenemase type in a isolate confirmed at the NRL as carrying a single enzyme.

Additionally, national phenotypic algorithms (https://www.argentina.gob.ar/sites/default/files/bancos/2021-10/Vigilancia-Nacional-de-la-Resistencia-a-los-Antimicrobianos-.pdf, accessed on 28 October 2024) for carbapenemase screening were retrospectively challenged with the susceptibility results obtained locally. In brief, this algorithm is based on specific phenotypic criteria for the suspicion of carbapenemases. For suspecting KPC or MBL-type enzymes in Enterobacteriaceae, the proposed criteria include non-susceptibility to imipenem or CZA [38,50,52]. Meanwhile, the Morganellaceae family relies on non-susceptibility to meropenem. Additionally, for suspecting OXA-type enzymes, the algorithm suggests non-susceptibility to ertapenem (EUCAST breakpoint) [38] and high-level resistance to piperacillin-tazobactam [53].

### 4.6. Statistics

Statistical analyses were performed in GraphPadPrism (version 10.2.3). Categorical variables were compared using Chi-square or Fisher’s exact test. Continuous variables were compared using a Wilcoxon rank-sum test. The Kolmogorov–Smirnov test was used to test for normal distribution of the data analyzed.

## 5. Conclusions

AMR challenges in Latin America have intensified post-COVID-19, with Argentina seeing a marked rise in multidrug-resistant infections, particularly from carbapenemase-producing Enterobacterales. This study underscores the need for ongoing surveillance and molecular characterization of resistance mechanisms. New data show both NDM and KPC carbapenemases circulate widely, with *K. pneumoniae* as the primary driver. Targeted interventions are essential to address regional resistance patterns, optimize treatments, and ensure access to last-resort antibiotics, curbing the spread of multidrug-resistant pathogens.

## Figures and Tables

**Table 1:** Distribution of species by source of isolation (n, % of the source of isolation for each species).

Species (Total of Isolates)	Urine	Blood	Respiratory Tract	Abdominal Tract	Others
Total (n 821)	344/821 (42)	208/821 (25)	97/821 (12)	40/821 (5)	132/821 (16)
*K. pneumoniae*	271/628 (43)	156/628 (25)	71/628 (11)	30/628 (5)	100/628 (16)
Morganellaceae ^1^	17/57 (30)	16/57 (28)	9/57 (16)	2/57 (3)	13/57 (23)
*E. cloacae*	16/51 (32)	12/51 (24)	11/51 (22)	0/51 (0)	12/51 (22)
*E. coli*	23/38 (60)	7/38 (18)	0/38 (0)	4/38 (11)	4/38 (11)
*Serratia* spp.	6/19 (32)	6/19 (32)	4/19 (21)	1/19 (5)	2/19 (10)
Other species ^2^	11/28 (39)	11/28 (39)	2/28 (7)	3/28 (11)	1/28 (4)

^1^ *Proteus mirabilis* (n 29), *Providencia stuartii* (n 25) and *Morganella morganii* (n 2). ^2^ *K. aerogenes* (n 14), *K. oxytoca* (n 7), *C. freundii* (n 6) and *C. koseri* (n 1). The cells highlighted in green indicate the site with the highest prevalence (*p* < 0.05).

**Table 2:** Distribution of carbapenemase molecular Classes and ESBL/plAmpC genes according to bacterial species.

Species	Number of Strains (%)	Number of Hospitals	Carbapenemase Class						CRE Non-CPE ^4^	ESBL/plAmpC Class			
			A	B	D	Combinations ^3^				CTX-M	CMY	PER	ESBL Combinations
						A + D	B + D	A + B/A + B + D					
Total	821 (100)	183	327 (39.8)	345 (42.0)	61 (7.4)	20 (2.4)	17 (2.1)	31 (3.8)	20 (2.4)	468 (57.0)	44 (5.4)	6 (0.7)	15 (1.8)
*K. pneumoniae*	628 (76)	142	276 (43.9)	263 (41.9)	33 (5.3)	16 (2.5)	7 (1.1)	25 (4.0)	8 (1.3)	391 (62.3)	20 (3.2)	2 (0.3)	11 (1.8)
Morganellaceae ^1^	57 (7)	28	1 (1.8)	39 (68.4)	7 (12.3)	1(1.8)	7 (12.3)	1(1.8)	1 (1.8)	15 (26.3)	11 (19.3)	0 (0)	1 (1.8)
*Enterobacter* spp.	51 (6)	40	21 (41.2)	12 (23.5)	11 (21.6)	1 (2.0)	1 (2.0)	1 (2.0)	4 (7.8)	29 (56.9)	0 (0)	2 (3.9)	1 (2.0)
*E. coli*	38 (5)	28	9 (23.7)	16 (42.1)	8 (21.1)	0 (0)	0 (0)	2 (5.3)	3 (7.9)	17 (44.7)	10 (26.3)	1 (2.6)	1 (2.6)
*Serratia* spp.	19 (2)	16	9 (47.4)	1 (5.3)	2 (10.5)	1 (5.3)	2 (10.5)	1 (5.3)	3 (15.8)	6 (31.6)	0 (0)	0 (0)	0 (0)
Other species ^2^	28 (4)	23	11 (39.3)	14 (50.0)	0 (0)	1 (3.6)	0 (0)	1 (3.6)	1 (3.6)	10 (35.7)	3 (10.7)	1 (3.6)	1 (3.6)

^1^ *Proteus mirabilis* (n 29), *Providencia stuartii* (n 25) and *Morganella morganii* (n 2). ^2^ *K. aerogenes* (n 14), *K. oxytoca* (n 7), *C. freundii* (n 6) and *C. koseri* (n 1). ^3^ Only NDM was detected in combinations. ^4^ CRE: Enterobacteral isolates with carbapenem resistance, non-carbapenemase producers (ESBL/AmpCplus impermeability). The cells highlighted in green indicate the most prevalent molecular mechanism (*p* < 0.05).

**Table 3:** Susceptibility profile of carbapenemase producers.

Carbapenemase Class	Number of Isolates (%)	Number of Hospitals	% Susceptibility													% Simultaneous Susceptibility to the Indicated Agents				
			CZA	IMR		AZA		FOS	TIG	AMK	COL	ATM		CFDC		FOS + COL	FOS + TIG	FOS + AMK	TIG + COL	AMK + TIG
				EUCAST	CLSI	EUCAST	CLSI					EUCAST	CLSI	EUCAST	CLSI					
Total	821 (100)	183	50.7	51.2	50.9	96.3	97.6	71.4	65.7	43.3	64.2	4.4	5.1	72.7	83.2	50.0	49.4	32.0	45.0	27.6
A	327 (39.8)	113	97.2	98.5	98.2	97.8	99.4	71.1	66.3	66.8	68.8	NA	NA	93.8	97.2	51.4	48.1	49.4	43.9	42.1
B	345 (41.9)	88	0	0	0	96.2	96.5	71.5	64.6	16.6	59.7	13.3	16.2	51.2	69.0	47.4	49.1	10.7	43.9	9.0
D	61 (7.4)	44	95.1	95.1	91.8	85.2	91.8	71.7	68.3	51.7	78.0	8.2	8.2	89.8	96.6	62.7	51.7	41.7	63.2	33.9
A + D	19 (2.3)	13	100	100	100	100	100	65.0	83.3	65.0	31.6	NA	NA	100	100	21.1	61.1	50.0	17.6	63.2
A + B. A + B + D ^1^	31 (3.8)	22	0	0	0	100	100	77.8	66.7	33.3	76.0	NA	NA	48.4	64.5	66.7	43.5	22.2	39.1	3.8
B + D	17 (2.1)	12	0	0	0	100	100	58.9	33.3	47.1	26.7	17.6	29.4	58.8	70.6	26.7	26.7	23.5	17.6	11.8
CRE non-CPE ^2^	20 (2.4)	17	100	100	100	95.2	95.2	81.0	71.4	81.0	75.0	20.0	20.0	NA	NA	60.0	57.1	61.9	61.9	57.1

^1^ Only NDM was detected in combinations. ^2^ CRE non-CPE: Enterobacteral isolates with carbapenem resistance, non-carbapenemase producers (ESBL/AmpC plus impermeability). CZA: ceftazidime-avibactam, IMR: imipenem-relebactam, AZA: aztreonam-avibactam, FOS: fosfomycin, COL: colistin, TIG: tigecicline; AMK: amikacin, CFDC: cefiderocol, ATM: aztreonam. NA: not applicable. The best-performing antibiotics or combination of antibiotics for treatment are shown in green.

**Table 4:** Susceptibility based on Enterobacterial species.

Species	Number of Strains (%)	Number of Hospitals	% Susceptibility												
			CZA	IMR		AZA		FOS	TIG	AMK	COL	ATM		CFDC	
				EUCAST	CLSI	EUCAST	CLSI					EUCAST	CLSI	EUCAST	CLSI
Total	821 (100)	183	50.7	51.2	50.9	96.3	97.6	71.4	65.7	43.3	64.2	4.4	5.1	72.7	83.2
*K. pneumoniae*	628 (76)	142	52.0	52.2	52.0	97.8	98.4	72.3	68.4	39.8	65.9	1.4	2.1	70.8	81.9
Morganellaceae ^1^	57 (7)	28	15.8	14.0	10.5	98.2	98.2	38.2	NA	33.3	NA	68.4	78.9	80.0	81.8
*Enterobacter* spp.	51 (6)	40	68.6	72.5	72.5	90.2	96.1	84.9	75.0	68.2	91.5	0.0	0.0	83.0	87.2
*E. coli*	38 (5)	28	50.0	52.6	52.6	78.9	84.2	88.6	96.9	53.8	94.3	13.2	18.4	79.4	97.1
*Serratia* spp.	19 (2)	16	78.9	78.9	78.9	100	100	82.3	50.0	73.7	NA	5.3	5.3	86.7	100
Other spp. ^2^	28 (4)	23	42.9	46.4	46.4	92.9	96.4	76.0	76.0	44.8	87.8	14.3	14.3	66.7	81.5

^1^ *Proteus mirabilis* (n 29). *Providencia stuartii* (n 25). *Morganella morganii* (n 2). ^2^ *K. aerogenes* (n 14), K. *oxytoca* (n 7), *C. freundii* (n 6), *C. koseri* (n 1). CZA: ceftazidime-avibactam, IMR: imipenem-relebactam, AZA: aztreonam-avibactam, FOS: fosfomycin, COL: colistin, TIG: tigecicline; AMK: amikacin, CFDC: cefiderocol, ATM: aztreonam, NA: not assessed. The best-performing antibiotics for treatment are shown in green.

## Data Availability

The data supporting the reported results are shown in Table S1.

## Supplementary Materials

The following supporting information can be downloaded at: https://www.mdpi.com/article/10.3390/antibiotics13121139/s1, Table S1. Bacterial species, site of isolation, resistance mechanisms, and susceptibility results of last resource antibiotics.
